# Herpetic Esophagitis in Immunocompetent Medical Student

**DOI:** 10.1155/2014/930459

**Published:** 2014-03-04

**Authors:** Andréia Vidica Marinho, Vinícius Mendes Bonfim, Luciana Rodrigues de Alencar, Sebastião Alves Pinto, João Alves de Araújo Filho

**Affiliations:** ^1^School of Medicine, Federal University of Goiás, Rua 235 com 1^a^ Avenida, Setor Universitário, 74605-020 Goiânia, GO, Brazil; ^2^Institute of Tropical Pathology and Public Health, Federal University of Goiás, Rua 235, s/n, Setor Universitário, 74605-050 Goiânia, GO, Brazil

## Abstract

Esophagitis caused by herpes simplex virus (HSV) is often documented during periods of immunosuppression in patients infected with human immunodeficiency virus (HIV); it is rare in immunocompetent diagnosed patients. Case reports of herpetic esophagitis in students of health sciences are extremely rare. The disease presents with a clinical picture characterized by acute odynophagia and retrosternal pain without obvious causes and ulcers, evidenced endoscopically in the middistal esophagus. Diagnosis depends on endoscopy, biopsies for pathology studies, and immunohistochemistry techniques. The disease course is often benign; however, treatment with acyclovir speeds the disappearance of symptoms and limits the severity of infection. In this report, we present a case of herpetic esophagitis in an immunocompetent medical student, with reference to its clinical features, diagnosis, and treatment. The disease may have manifested as a result of emotional stress experienced by the patient.

## 1. Introduction

Esophagitis caused by herpes simplex virus (HSV) is frequently documented during periods of immunosuppression in patients infected with human immunodeficiency virus (HIV). This condition can also occur as a primary infection in individuals taking immunosuppressive drugs and it is therefore considered as an opportunistic disease [1].

Cases of herpetic esophagitis in young immunocompetent individuals are rare in the literature; reports in which the patient is a healthcare student are even more unusual.

Although herpetic esophagitis is rare in immunocompetent individuals, it should be considered as a diagnostic hypothesis for clinical conditions characterized by acute odynophagia and retrosternal pain without other obvious causes and ulcers, evidenced endoscopically in the mid-distal esophagus [1].

Thus, the aim of this case report is to describe esophagitis caused by herpes simplex virus in an immunocompetent, healthy, female medical student.

## 2. Case Report

A 22-year-old, white, single female medical student reported pain when swallowing. The pain manifested four days prior to presentation and began with a burning epigastric pain and intense heartburn. The patient was self-medicated with omeprazole and domperidone without success. After approximately 12 hours, there was a change in the pattern of pain, which was constrictive and intermittent and was located in the sternal region.

The patient developed asthenia, malaise, appetite loss, and a daily fever of up to 38.5°C during the afternoon/early evening. She reported a weight loss of 5 kg over one week with associated nausea and odynophagia for solids, pastes, and liquids. She initially experienced pain only in the distal esophagus, which later expanded to the full extension of the esophagus.

A physical examination revealed mild hyperemia in the oropharynx, good general condition and mucous with normal coloration. The patient was hydrated, afebrile, acyanotic, and anicteric. Her respiratory system, cardiovascular system, and abdomen were unchanged. Additionally, she had no cervical, axillary, or inguinal lymphadenopathy.

The tests performed on the patient are listed in the board table, and the results are provided in Table 1.

**Figure 1:**
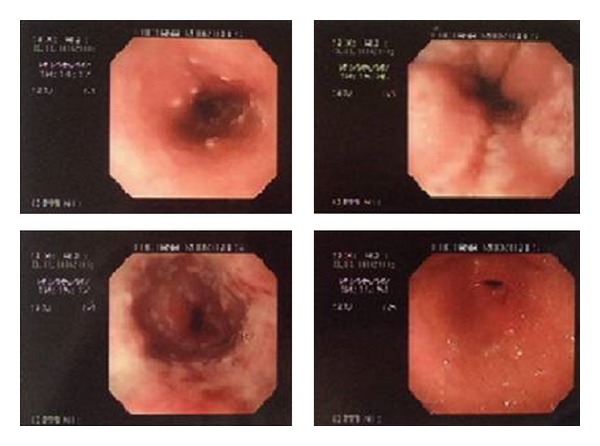
Upper digestive endoscopy showing numerous injuries on the esophageal surface that were yellow-whitish in color, pleomorphic, and isolated small circular plaques with central erosions and even depression with hyperemia at baseline; these injuries were more prominent in the distal third of the esophagus, indicating infectious esophagitis.

The following observations were made during upper digestive endoscopy (Figure 1): numerous injuries on the esophageal surface that were yellow-whitish in color, pleomorphic and isolated in small circular plaques with central erosions and even depression with hyperemia at baseline; these injuries were more prominent in the distal third of the esophagus, indicating infectious esophagitis.

**Figure 2:**
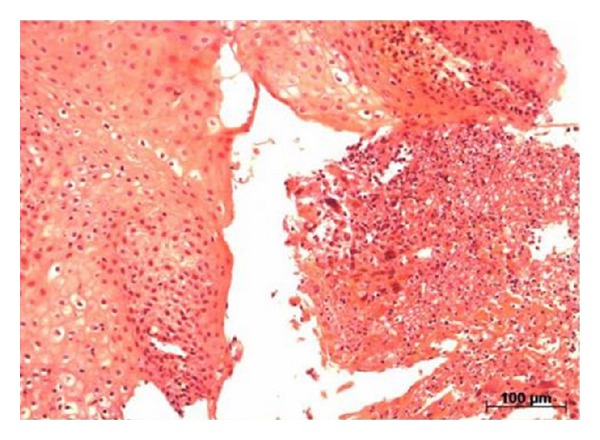
Anatomic pathological examination of the esophagus showing ulceration with presence of multinucleated cells with overlapping nuclei and ground-glass aspect, consistent with herpetic infection (hematoxylin-eosin).

During the anatomic pathological examination of the esophagus, the esophageal mucosa showed ulceration with the presence of multinucleated cells with overlapping nuclei and ground-glass aspect, which is consistent with herpetic infection (Figure 2).

**Figure 3:**
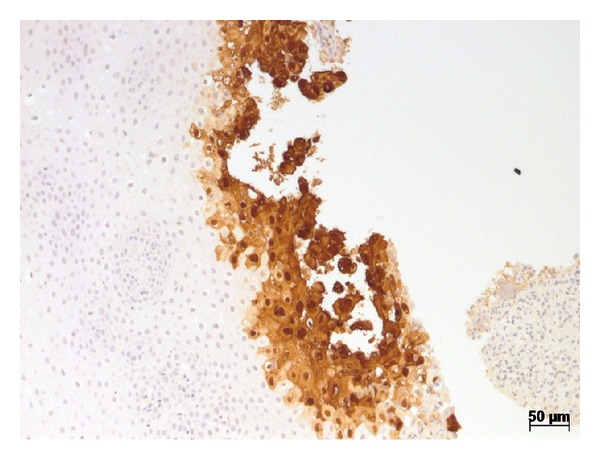
Immunohistochemical study of the esophagus's fragment showing positivity to herpes simplex virus type 1.

An immunohistochemical study of the patient's esophagus revealed that the patient was positive for herpes virus type 1 (Figure 3). The assay utilized was a polyclonal rabbit antiherpes simplex virus type 1 assay (Dako, Albertslund, Denmark).

The patient was treated with oral acyclovir (800 mg) 5 times a day for 7 days. Early improvement was noted on the third day, and complete resolution of symptoms was achieved in 1 week.

## 3. Discussion

The incidence of HSV in immunocompromised patients, such as HIV carriers, organ transplant recipients, patients with neoplasias, and individuals treated with corticosteroids or immunosuppressive drugs, is high. Additionally, HSV has a range of manifestations, from asymptomatic infections to fatal disease. Herpetic esophagitis is often diagnosed in immunocompromised patients but can also occur in immunocompetent patients [2].

Herpetic esophagitis appears to be more common in men, with a ratio of 3 affected men for every 1 woman; however, in the present report the patient is female. Initial clinical manifestations include odinophagy and heartburn, and prodromes include fever, sore throat, and respiratory symptoms. Simultaneous oropharyngeal or genital lesions are reported as symptoms in only 20% of cases [3]. Other symptoms previously published in the literature include acute onset of esophageal complaints such as chest pain (46.4%), odinophagy (60.7%), dysphagia for both solids and for liquids (37.5%), heartburn, and/or vomiting [4].

Before the appearance of the upper digestive symptoms, the clinical course includes nonspecific symptoms of flu with a temperature of 39°C, malaise, anorexia, and weight loss. In this case, the patient presented with asthenia, poor general condition, appetite loss, and a daily fever of up to 38.5°C during the afternoon or early evening [5].

Occasionally, the coalescence of ulcers in the lower third of the esophagus may resemble severe peptic esophagitis. Thus, it is important to have a proper clinical suspicion to guide the biopsy and culture of the esophageal mucosa [6].

The diagnosis of herpetic esophagitis is usually made by upper digestive endoscopy, which often reveals the extensive involvement of the disease with numerous ulcers and whitish exudates [3]. Additionally, the endoscopy may reveal aspects of injury and allows tissue sampling for histology and viral culture [7].

A pathological examination can provide cytopathologic and immunohistochemical characteristics that are useful for confirming the disease. Both polymerase chain reaction (PCR) and direct immunofluorescence assays (DFA) are viable in these cases. DFA studies can provide faster results; however, they have a sensitivity of only 69–88%, whereas PCR tests have a sensitivity of 92–100% with 100% specificity. Finally, viral cultures obtained from suspicious lesions observed on upper digestive endoscopy can confirm the diagnosis [8].

However, the endoscopic appearance of herpetic esophagitis can be confused with eosinophilic esophagitis or with esophagitis caused by *Candida* sp. or cytomegalovirus (CMV) [9].

Negative results were obtained by the immunoassay method for both IgM and IgG against herpes simplex (Table 1). This result may have occurred because the current case is a primary infection or because the serological test was performed early (before the production of antibodies). The clinical diagnosis was based on positive histopathology and immunohistochemistry.

It is believed that herpetic esophagitis is self-limiting in immunocompetent patients. Spontaneous healing may occur after several weeks; however, case reports suggest that recovery can be accelerated by treatment with acyclovir or valacyclovir. Although there are no clinical studies documenting the benefits of antiviral therapy in immunocompetent hosts, their benefit in immunocompromised patients has been clearly documented [6].

Among the reported cases of adults who received acyclovir therapy, most achieved a clinical response within 24–72 hours, and all became asymptomatic within 4 to 14 days without complications [3, 10]. These observations suggest that antiviral therapy in immunocompetent hosts can accelerate the resolution of symptoms and can prevent complications.

Finally, the clinical physiopathology of primary HSV infection is not well understood, although it is known that stress may be an important immunosuppressive factor [11].

Importantly, the fact that the patient was in a situation of emotional stress due to her mother's health and her college assessments may have contributed to the manifestation of symptomatic esophageal herpes, although studies have demonstrated that the link between psychosocial stress and oral herpes is stronger than that between stress and esophageal or vaginal herpes [12].

Additionally, it is believed that the association between psychosocial stress and symptomatic manifestation of the disease is stronger in the female population; therefore, women are more vulnerable to herpes influenced by psychological effects than men [12].

Herpetic esophagitis should be taken into consideration in immunocompromised patients. However, it is very important to clarify that although herpetic esophagitis is a rare disease in immunocompetent patients, it is vital to investigate the possibility that this disease might exist in such patients and to include it in the differential diagnosis, even in healthy patients.

## Figures and Tables

**Table 1:** Tests performed on the patient.

Exam	Result
Erythrocytes	4,7 million/mm^3^
Hemoglobin	14,2 mg/dL
Hematocrit	41%
Leukocytes	9.700/mm^3^
Rod-shaped	2%
Segmented	58%
Eosinophils	3%
Lymphocytes	35%
Monocytes	2%
Basophils	0%
Thrombocytes	204.000/mm^3^
Urea	40 mg/dL
Creatinine	0,7 mg/dL
Fasting blood glucose	85 mg/dL
SGPT/ALT	40 UI/L
SGOT/AST	35 UI/L
Cytomegalovirus (antibodies)	
IgM	Not reagent
IgG	Reagent
Herpes simplex I/II (antibodies)	
IgM	Not reagent
IgG	Not reagent
HIV I/II (antibodies)	Not reagent

Legend:

SGPT/ALT: serumglutamicpyruvic transaminase/alanine transaminase.

SGOT/AST: serumglutamicoxaloacetic transaminase/aspartate transaminase.

IgM: immunoglobulin M.

IgG: immunoglobulin G.
